# An Efficient and Secure Data Sharing Method Using Asymmetric Pairing with Shorter Ciphertext to Enable Rapid Learning in Healthcare

**DOI:** 10.1155/2022/4788031

**Published:** 2022-04-20

**Authors:** Snehlata Yadav, Namita Tiwari

**Affiliations:** Department of Computer Science and Engineering, Maulana Azad National Institute of Technology, Bhopal 462003, India

## Abstract

The recent advent of cloud computing provides a flexible way to effectively share data among multiple users. Cloud computing and cryptographic primitives are changing the way of healthcare unprecedentedly by providing real-time data sharing cost-effectively. Sharing various data items from different users to multiple sets of legitimate subscribers in the cloud environment is a challenging issue. The online electronic healthcare system requires multiple data items to be shared by different users for various purposes. In the present scenario, COVID-19 data is sensitive and must be encrypted to ensure data privacy. Secure sharing of such information is crucial. The standard broadcast encryption system is inefficient for this purpose. *Multichannel broadcast encryption* is a mechanism that enables secure sharing of different messages to different set of users efficiently. We propose an efficient and secure data sharing method with shorter ciphertext in public key setting using asymmetric (Type-III) pairings. The Type-III setting is the most efficient form among all pairing types regarding operations required and security. The semantic security of this method is proven under decisional BDHE complexity assumption without random oracle model.

## 1. Introduction

Cloud computing is a new paradigm of computing system that has revolutionized many sectors of Government and corporate such as academic, healthcare, online social networking, banking, and automobile. To enhance productivity, all these sectors consider data sharing as a vital tool to overcome the time and location constraints of resource usage such as computing power or data storage according to the need of users. Cloud computing environment provides large storage capacity and strong computation power. Thus, it brings ultimate convenience to the legitimate users. The outstanding advantage of cloud computing is that cloud service users can use their computing resources as a service with minimal cost at any time through the Internet that transcends geographical limits. Software as a Service (SaaS), Platform-as-a-Service (PaaS), Infrastructure-as-a-Service (IaaS) and Data-as-a-Service (DaaS) [1] are the four major services offered by cloud. Different cloud models support different services. There are many advantages in cloud, one of which is virtualization. Virtualization is also one of the strong pillars of cloud computing. Cloud computing system helps multiple users across the world to share and exchange their data in secure manner. Data sharing service is regarded as the most exciting use-case of cloud storage system, which has become the most important area in cloud computing. Apple's iCloud, Microsoft's Azure [2], and Amazon's S3 [3] are renowned for offering a more flexible and easy way to share data over the Internet. Despite this, they are susceptible to various security threats, which are the primary concerns of cloud users [2]. Security threats from external adversary are a bit obvious. However, nowadays, data owners outsource their data in the cloud server and want to share these data securely with other legitimate cloud users; various cryptography techniques can be adopted to enhance the secure exchange of data among subscribed users.

### 1.1. Problem Formulation

**Figure 1:**
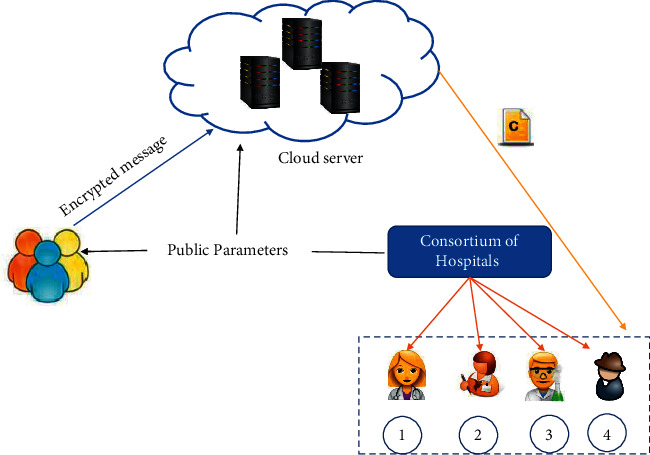
A system model for the e-healthcare scenario.

Consider an online e-healthcare system (Figure 1) where the data of patients such as COVID-19 data and OPD data from various data owners (doctors from several hospitals) are collected and uploaded to the centralized storage server, say cloud server, in encrypted form for security perspective, using a key, given by some authority such as hospital consortium. This is an example of data in transit. If this issue is not taken into consideration, the patients may suffer from enormous consequence of information leak. Recently millions of user data have been compromised. As per Government guidelines, there is a necessity to keep COVID-19 data private and secure.

**Figure 2:**
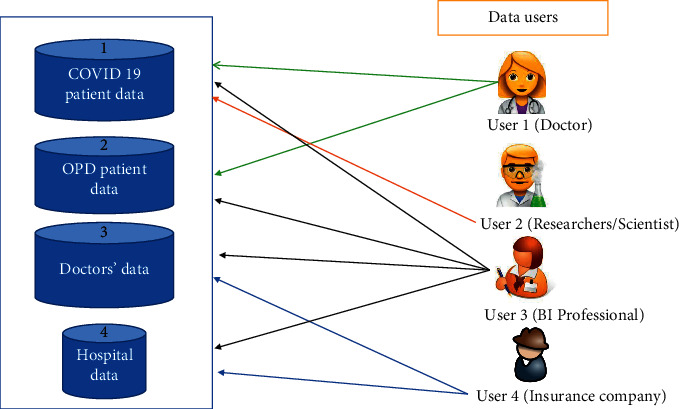
A MCBE-based system model for online data sharing.

In addition to COVID-19 and OPD data of patients, online healthcare system consists of doctors data, healthcare workers data, hospital data, pharmaceutical data, and so forth. Such crucial data uploaded in encrypted form are supposed to be analyzed and used by different legitimate data users; for example, due to this pandemic, a doctor could use information of patients to provide treatment and follow-up remotely, and a researcher/scientist at the research center could analyze patients record to find new symptoms appearing in patients. Based on their observation, they come up with the solutions and prevention methods. A business intelligence professional (BIF) could analyze patients records to generate the visualization of periodic health analysis report. A patient could search for a doctor (specialist) of their interest for better healthcare. An insurance company could use hospital data and pharmaceutical data for mediclaim disbursement and so forth. To accomplish all these tasks, the encrypted data stored on the server must be shared in efficient and flexible manner. However, an online healthcare system consists of multiple disjoint entities for data generation and data access. Since online healthcare data most of the time resides in shared environments, ensuring sharing and accessing the data securely on the cloud is a nontrivial task. One way to share data among a group of legitimate subscribers is broadcast encryption. Transmitting data to many groups of subscribers needs multiple instances of broadcast encryption which is highly inefficient. Multichannel broadcast encryption (Figure 2) is the efficient solution for sharing multiple data among multiple groups of legitimate subscribers in the cloud environment.

For example, we assume that our system model has 4 databases (*m* = 4): COVID-19 patient data, OPD patient data, doctors' data, and hospital data. The maximum number of subscribers for each database is 50 (*n* = 50). Assume that Alice (User1) is doctor, Bob (User2) is a researcher or scientist, Kim (User3) is a BI professional, and Ram (User4) is an officer from Insurance Company. If Dr. Alice is required to share COVID-19 patient data and OPD patient data, she subscribes to databases 1 and 2 and receives the corresponding decryption keys by hospital consortium. Bob wants to access COVID-19 data; he subscribes to database 1. Kim requires COVID-19 patient data, OPD patient data, doctors' data, and hospital data for making dashboards; she subscribes to databases 1, 2, 3, and 4. Ram requires doctors' data and hospital data for mediclaim and so forth; he subscribes to databases 3 and 4. The broadcaster encrypts data for the subscribers Alice, Bob, Kim, and Ram, using public parameters provided by hospital consortium. The broadcaster creates four target sets as follows:

- Set *S*_1_ corresponding to database 1 (COVID-19 patient data), which is intended for Alice (User1), Bob (User2), and Kim (User3). The session key would be *K*_1_. The legitimate subscribers are given as *S*_1_  = {User1,User2,User3}.
- Set *S*_2_ corresponding to database 1 (OPD patient data), which is intended for Alice (User1) and Kim (User3). The session key would be *K*_2_. The legitimate subscribers are given as *S*_2_  = {User1,User3}.
- Set *S*_3_ corresponding to database 1 (doctors' data), which is intended for Kim (User3) and Ram (User4). The session key would be *K*_3_. The legitimate subscribers are given as *S*_3_  = {User3, User4}.
- Set *S*_4_ corresponding to database 1 (hospitals data), which is intended for Kim (User3) and Ram (User4). The session key would be *K*_4_. The legitimate subscribers are given as *S*_4_  = {User3,User4}.

In the above example, we have four subsets *S*_1_, *S*_2_, *S*_3_, *S*_4_. Thus, here targeted set of subscribers *t*  = 4, where *t* ≤ *m*. The detailed mathematical description of the scheme is presented in Section 3.

Another scenario could be an online academic system where online exam papers are distributed by some central authority. Let us take an example of Language paper; suppose that central authority of exam has to take 28 language papers corresponding to various states. The authority wants to send these exam question papers to authorised exam centers in a secure way. Multichannel broadcast encryption provides an efficient way for solving this problem. There are many real-time cases where multichannel broadcast encryption can be applied.

In this paper, we propose an efficient method of data sharing by multiple different users to multiple different legitimate subscribers in a secure and flexible way. The major contribution is listed as follows:

- Multichannel broadcast encryption scheme [4] is based on the setting of symmetric pairings. Type-I setting is slower as compared to Type-III setting [6]. The proposed scheme is constructed in Type-III setting. It is of interest to convert MCBE construction from symmetric to asymmetric bilinear pairings [5]. The asymmetric variant is definitely faster and efficient and has compact implementation, which will arise from the benefit over the symmetric setting.
- Most of the schemes available in literature are in private key setting but the proposed scheme is in public key setting and has a small ciphertext size.
- The semantic security of the scheme is based on Decisional Bilinear Diffie-Hellman Exponent (DBDHE) hardness assumption.
- The proposed construction achieves selective security in the random oracle model (ROM).

The rest of the manuscript is organized as follows: Section 2 covers mathematical notations and computational complexity assumptions on which broadcast encryption schemes are constructed. The framework of conversion from symmetric setting to asymmetric setting is described in Section 3. In Section 4, the security model and correctness proof of conversion to asymmetric pairing are covered. The proposed scheme is then analyzed based on scheme complexity in Section 5.  Section 6 concludes the paper including some open problems.

### 1.2. Related Work

**Figure 3:**
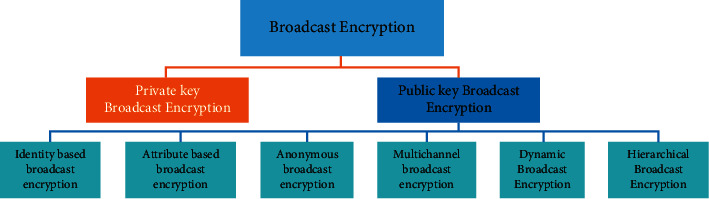
Categorization of broadcast encryption schemes.

Broadcast encryption [6] is a useful cryptographic primitive and has been widely studied as it is the fundamental primitive for many real-life applications. It was introduced by the seminal work of Fiat and Naor in year 1993, but it received much attention after the realization of Naor, Naor, and Lotspiech scheme [7]. Broadcast encryption cryptographic primitive provides a solution to the problem of communicating an encrypted message to only set *S* of legitimate users over insecure public channel. In more detail, users who get access to ciphertext are called privileged subscribers. They are members of set *S* and nonmembers of *S* are called revoked users. Thus, the broadcast algorithm is considered to work on the partition of revoked and legitimate users and the partition may vary for each broadcast message. Revoked users cannot learn a single bit of encrypted message even if they collude in some way. This property is called collusion resistance property. Due to this, broadcast encryption (BE) [8] has potential applications in fields such as pay TV, satellite TV, encrypted mailing services, and encrypted file system in cloud applications. Broadcast encryption is deployed in two ways based on keys, namely, symmetric-key setting and asymmetric-key setting. In symmetric-key setting, a key generation center distributes the secret decryption key to all legitimate users in advance even before the message is transmitted. In such a scenario, only broadcaster acts as an emitter of message. The plaintext is encrypted by the emitter using a session key and in turn the session key is encrypted using the keys of the legitimate users of set *S*. So, for every new broadcast message, if new user joins and existing user leaves the system, the secret key has to be refreshed. Modifying and refreshing key, when at least one user leaves or joins in the system, is called one-affects-all problem. The problem is efficiently addressed by the broadcast encryption in public-key (asymmetric-key) setting. In this kind of setting, all users of *S* have a pair of keys: encryption key and decryption key. Broadcaster and other entities can act as emitter; however, only legitimate subscribers can decrypt the message and read the actual plaintext. It also alleviates the problem of refreshing the secret keys when a new member joins the system. The secure transmission of secret keys to all users of system has a problem of key compromise by members of *S*. Broadcast encryption is put forth by the seminal work of Fiat and Naor [6] followed by many constructions that have been proposed in [9] with different objectives of reducing decryption key size, encryption key size, encrypted message size, and computational cost of construction. Broadcast encryption in public-key setting is well studied and further categorized in Figure 3 as follows: identity-based broadcast encryption, attribute-based broadcast encryption, anonymous broadcast encryption, hierarchical broadcast encryption, dynamic broadcast encryption, and distributed broadcast encryption. Thus, it has many practical applications such as secure e-mail system, digital rights management system, pay TV, database security system, online social network system, and blockchain. Waters and Sahai [10] realized an extension of identity-based encryption which was later named as attribute-based encryption, in which inspite of identity as a public key, attributes of legitimate recipients are used for encrypting messages. ABE constructions' major problem is collusion resistance and recipient revocation. In some circumstances, one may want to give access right to a subset of recipients rather than only one specific recipient; to facilitate this, the notion of attribute-based broadcast encryption [11] has been realized. Figure 3 represents the broad categorization of broadcast encryption variants of it in public key framework.

1. Identity-based broadcast encryption: the notion of identity-based broadcast encryption (IBBE) scheme was first introduced by Delerablée [12]. It is an extension of identity-based encryption scheme in public-key setting where, instead of public keys of the legitimate users, their identity, such as an e-mail id, passport number, and driving license number (strings of characters, alphanumeric values, and numerals), was used as encryption key to encode the message. IBBE is a practical cryptographic primitive that allows exponential number of recipients to exchange messages in secure manner; this implies that the public parameters are not correlated to decryption key of recipients and to ciphertext transmitted among subscribers. The first optimal IBBE scheme [13] has been constructed from pairings and learning with errors (LWE).
2. Attribute-based broadcast encryption: Sahai and Waters [10] realized an extension of fuzzy identity-based encryption which was later termed as attribute-based encryption. In this, despite identity being a public key, attributes of legitimate recipients have been taken into account for encrypting messages which can be decrypted by a set *S* of subscribers, that is, those who belong to attribute set. ABE schemes suffer from the problem of collusion resistance and recipient revocation. Some scenario requires to provide access right to a subset of recipients rather than only one specific recipient. To facilitate this, the notion of attribute-based broadcast encryption [11] has been realized. ABBE has been well studied by the research community in recent years [14] which includes various hardness assumptions such as bilinear map, multilinear map, LWE, and R-LWE. LWE and R-LWE constructions are quantum-resistant but are not good candidates for resource-constrained environment as key size and ciphertext size become large for light weight devices [14].
3. Anonymous broadcast encryption: in the standard broadcast encryption (BE) cryptosystem, recipients' information is revealed from the encrypted message. This is also considered as a security gap since it enables automatic disclosure of identity. However, many BE scenarios demand to hide the target identity, as the identity also conveys sensitive information and can cause identity threat, if it gets disclosed. The notion of anonymous BE gets rid of this and enables users to search on encrypted data. In year 2006, Barth et al. [15] introduced another variant of broadcast encryption (BE), known as *anonymous broadcast encryption* (Ano-BE) which is chosen-ciphertext-attack- (CCA-) secure in random oracle model (ROM). Subsequently, [16–18] have shown enhancement on this primitive.
4. Dynamic broadcast encryption: in SCN 2012, Phan et al. [19] first introduced a primitive of BE called dynamic decentralized BE (D-BE). In the traditional broadcast encryption system, a central authority was responsible for management of a set of subscribers. To decentralize such a system, D-BE primitive uses subset cover framework with DDH hardness assumption.
5. Hierarchical broadcast encryption: this variant of BE is constructed on pairing based cryptographic primitive that enables key delegation property to subsequent descendants in the hierarchical system. This was first proposed by [20] for identity-based BE scheme. The later scheme is IND-CCA secure with constant ciphertext size in standard model.
6. Functional broadcast encryption: this variant of BE enables access control along with public-key cryptography for sending encrypted file to specific subset of subscriber [21]. This scheme is based on indistinguishability obfuscation and achieves selective IND-CCA security.
7. Multichannel broadcast encryption: this variant of BE enables sending an encrypted message to different groups of users. Consider a scenario of secret space program where the scientists of various states of a country are working together and the project coordinator wants to transmit different kinds of encrypted data to the various teams located in different geographical locations simultaneously. Multichannel broadcast encryption scheme was first presented by [4] in *ASIA CCS 13.* The scheme was designed in symmetric-key setting and achieves chosen-plaintext (CPA) and chosen-ciphertext security in standard model. It was further modified by [22]. In CANS 2018, [16] designed the scheme in public-key framework using decisional BDHE-sum assumption. The scheme has constant header size and achieves selective security. Acharya's [23] one construction achieves semistatic security and another construction achieves selective security with high computation cost. Both schemes are constructed in Type-I pairing [5]. Very recently, Le et al. [24] have constructed a scheme using GDDHE hardness assumption in public-key setting. The scheme achieves selective security in random oracle model.

Cloud is the most promising platform to share health related data. Online e-healthcare models [25, 26] are deploying cloud for sensitive data sharing. Many broadcast encryption primitives [27, 28] have been used for data sharing in cloud environment. These primitives are available in private- as well as public-key setting [29]. As far as our problem is concerned, we are interested in multichannel broadcast cryptographic primitives that allow sharing of different messages to different users. Most of the constructions are in private-key setting and Type-I pairing. However, these schemes suffer from the limitations of private-key settings as well as Type-I pairing setting.

## 2. Preliminaries

### 2.1. Notations

We introduce same notations as presented in [4]. The notations are summarized in Table 1. For a set *B*, let $b\overset{R}{\leftarrow }B$ indicate that *b* is a uniformly selected random element from set *B*. In the following, we will assume that there exists an asymmetric bilinear map $\hat{e}:{𝔾}_{1}\times {𝔾}_{2}⟶{𝔾}_{T}$, where *𝔾*_1_=〈*P*〉 and *𝔾*_2_=〈*Q*〉 are groups of the elliptic curve of the same prime order *q* with generators *P* and *Q*, respectively. As both groups are of prime order, any nonidentity elements of *𝔾*_1_ and *𝔾*_2_ are the generators of the group. Finally, any element in group *𝔾*_1_, *𝔾*_2_, or *𝔾*_*T*_ is assumed to have size *𝒪*(*η*_1_), *𝒪*(*η*_2_), *𝒪*(*η*_*T*_), respectively.

Let *α* be a uniformly chosen random element of *ℤ*_*q*_. For any element *N* from either *𝔾*_1_ or *𝔾*_2_, let *N*_*x*_=*α*^*x*^*N*, where *x* ∈ *ℤ*_*q*_.

Consider ${\overset{⟶}{Y}}_{N,\alpha ,l}=\left\{N_{1},N_{2},\ldots ,N_{l},N_{l+2},\ldots ,N_{2l}\right\}$ as a set of 2*l* − 1 elements. The term *N*_*l*+1_ is not included in ${\overset{⟶}{Y}}_{N,\alpha ,l}$, so that the bilinear pairing would be of a little help in evaluating $\hat{e}{\left(P,Q\right)}^{\alpha^{l+1}}$.

### 2.2. Bilinear Map Based on Prime Order Groups

Let *𝔾*_1_ and *𝔾*_2_ be two additive groups of same prime order and let *𝔾*_*T*_ be multiplicative cyclic group of prime order *q* for some large prime *q*. *P* is a generator of *𝔾*_1_ and *Q* is a generator of *𝔾*_2_; pairing is defined as a function $\hat{e}:{𝔾}_{1}\times {𝔾}_{2}⟶{𝔾}_{T}$ [30].

A pairing is defined to be admissible if it satisfies the following properties:

1. Bilinearity: $\hat{e}\left(aP,\text{bQ}\right)=\hat{e}{\left(P,Q\right)}^{ab}=\hat{e}\left(bP,aQ\right)$, ∀*P* ∈ *𝔾*_1_, ∀*Q* ∈ *𝔾*_2_, and ∀*a*, *b* ∈ *ℤ*_*q*_.
2. Nondegeneracy: $\hat{e}\left(P,Q\right)$ is a generator element of *𝔾*_*T*_; that is, ${𝔾}_{T}=\langle \hat{e}\left(P,Q\right)\rangle$, where $\hat{e}\left(P,Q\right)\neq 1$.
3. Computability: a pairing is defined as computable if there exists an algorithm that can compute $\hat{e}\left(P,Q\right)$, ∀*P* ∈ *𝔾*_1_, ∀*Q* ∈ *𝔾*_2_, and ∀*a*, *b* ∈ *ℤ*_*q*_ efficiently. There are three types of bilinear maps [31, 32] used in the construction of various pairing-based schemes:

- If *𝔾*_1_=*𝔾*_2_, the pairing is termed as *symmetric pairing* or *Type-I* pairing
- If *𝔾*_1_ ≠ *𝔾*_2_ and there exists an efficiently computable homomorphism *ϕ* : *𝔾*_2_⟶*𝔾*_1_, the pairing is referred to as *Type-II* pairing
- If *𝔾*_1_ ≠ *𝔾*_2_ and there does not exist an efficiently computable homomorphism *ϕ* : *𝔾*_2_⟶*𝔾*_1_, the pairing is referred to as *asymmetric pairing* or *Type-III* pairing

### 2.3. Computational Complexity Assumption

In this section, the computational complexity assumption of multichannel broadcast encryption scheme is introduced. The symmetric and asymmetric versions of decisional BDHE assumption are proposed [28, 33].

#### 2.3.1. Bilinear Diffie–Hellman Exponent (BDHE) Assumption in the Symmetric Pairing Setting

Let *P* and *Q* be two random generators of cyclic group *𝔾* of prime order *q* and $\alpha \overset{\left[R\right]}{\leftarrow }{\mathbb{Z}}_{p}$ such that *Q*_*i*_=*Q*^*α*^*i*^^. The n-BDHE problem in *𝔾* is defined as follows:

  Let ${\overset{⟶}{Y}}_{Q,\alpha ,n}=\left(Q_{1},\ldots ,Q_{n},Q_{n+2},\ldots ,Q_{2n}\right)$
  Input instance: $\left(P,Q,{\overset{⟶}{Y}}_{Q,\alpha ,n},D\right)$
  Output: $D=\hat{e}\left(Q_{n+1},P\right)=\hat{e}{\left(P,Q\right)}^{\alpha^{n+1}}\in {𝔾}_{T}$

An algorithm *𝒜* has advantage *ϵ* in solving n-BDHE problem in *𝔾* if Pr[*𝒜*(*P*, *Q*, *Q*_1_,…, *Q*_*n*_, *Q*_*n*+2_,…, *Q*_2*n*_)=*D*] ≥ *ϵ*, where the probability is over the random choices of generator (*P*, *Q*) ∈ *𝔾*, random choice of *α* ∈ *ℤ*_*p*_, the random choice of *D* ∈ *𝔾*_*T*_, and the random bits *β* ∈ {0,1} used by *𝒜*.

**Definition 1 .**

The decisional (*τ*, *ϵ*, *l*)-BDHE hardness assumption holds in *𝔾* if no *τ*-time algorithm has advantage at least *ϵ* in solving the *l*−BDHE problem in *𝔾* [33].

#### 2.3.2. Bilinear Diffie–Hellman Exponent (BDHE) Assumption in the Asymmetric Pairing Setting

Security of multichannel broadcast encryption schemes in asymmetric bilinear pairing is based on the well-studied complexity assumption known as Bilinear Diffie-Hellman Exponent (BDHE) assumption [33]. Consider two bilinear groups *𝔾*_1_ and *𝔾*_2_ of same prime order *q*. Given *P*, *Q*, and *α*^*i*^*P*=*P*^*α*^*i*^^ in either *𝔾*_1_ or *𝔾*_2_, for *i*=(1,2,…, *l*, *l*+2,…, 2*l*), ${\overset{⟶}{Y}}_{P,\alpha ,l}=\left(P_{1},P_{2},\ldots ,P_{l},P_{l+2},\ldots ,P_{2l}\right)$.

The asymmetric decisional *l*−BDHE problem is defined as follows.

Input instance: $\left(ℋ,P,Q,{\overset{⟶}{Y}}_{P,\alpha ,l},{\overset{⟶}{Y}}_{Q,\alpha ,l},D\right)$.

Output: $D=\hat{e}\left(P_{l+1},ℋ\right)\in {𝔾}_{T}$.

Since the term *l*+1 is missing from the sequence of powers, the bilinear map appears to be of no help in computation of $\hat{e}\left(P_{l+1},ℋ\right)$. The bilinear pairing $\hat{e}$ decides whether $D=\hat{e}\left(P_{l+1},\;ℋ\right)$ holds or not. As a shorthand, once *P*, *Q*, and *α* are specified, *y*_*i*_ is set as *y*_*i*_=*α*^*i*^*y*=*y*^*α*^*i*^^, where *y* is in either *𝔾*_1_ or *𝔾*_2_.

Let *Adversarial* algorithm *𝒜* be a *τ*-time algorithm that receives an input challenge for asymmetric *l*−BDHE problem and produces a decision bit *β* ∈ {0,1} as output. *𝒜* has advantage *ϵ* in solving asymmetric decisional *l*−BDHE problem when the difference between $\left|\text{Pr}\left[𝒜\left(ℋ,P,Q,{\overset{⟶}{Y}}_{P,\alpha ,l},\hat{e}\left(P_{l+1},\;ℋ\right)\right)=0\right]\right|$ and $\left|\text{Pr}\left[𝒜\left(P,Q,{\overset{⟶}{Y}}_{P,\alpha ,l},\hat{e}\left(P_{l+1},\;ℋ\right)=0\right)\right]\right|$ is ≥*ϵ*, where the probability is over the random choices of *D* ∈ *𝔾*_*T*_, random bits consumed by *𝒜*, random choice of *α* ∈ *ℤ*_*q*_, and the random choices of generators *P* and *Q* of *𝔾*_1_ and *𝔾*_2_, respectively.

**Definition 2 .**

*The asymmetric decisional*(*τ*, *ϵ*, *l*)*-BDHE hardness assumption holds in*(*𝔾*_1_, *𝔾*_2_)*if noτ-time algorithm has advantage at leastϵin solving the asymmetricl*−*BDHE problem in*(*𝔾*_1_, *𝔾*_2_) [28].

### 2.4. Multichannel Broadcast Encryption

Multichannel broadcast encryption (MCBE) is a variant of broadcast encryption introduced by [4] inspired by the construction of [34]. In this cryptosystem, a Private-Key Generation Centre (PKGC) generates decryption keys and global public parameters. A broadcaster generates ciphertexts {*C*_*i*_}_*i*=1_^*m*^ corresponding to a message *M* for *m* disjoint groups of legitimate users {*G*_*i*_}_*i*=1_^*m*^. A legitimate user *u* ∈ *G*_*i*_ retrieves the plaintext *M* using own decryption key. The description of MCBE cryptographic primitive scheme is as follows.

#### 2.4.1. Syntax of MCBE

An MCBE scheme is four-tuple of algorithms: MCBE =(Setup, KeyGen, Enc, Dec).

1. (Param, MSK) ← Setup(*N*, *λ*): *Setup* is also known as PKGC which takes as input maximum count of users *N* accumulated in the system and security parameter *λ*. The PKGC outputs the public parameter Param and a master secret key MSK. Param is made public for all and MSK is kept secret.
2. *d*_*u*_ ← KeyGen(Param, MSK, *u*): it takes Param, MSK, and a legitimate user *u* as inputs and produces a decryption key *d*_*u*_ corresponding to user *u* as output. *d*_*u*_ is sent to *u* over a secure communication link established between PKGC and the legitimate user *u*.
3. (*𝒞*, {*K*_*i*_}_*i*=1_^*m*^) ← Enc(*S*_1_, *S*_2_,…, *S*_*m*_, Param): it takes input Param and set of legitimate users {*S*_*i*_}_*i*=1_^*m*^, with each *S*_*i*_⊆*G*_*i*_. The broadcaster entity outputs a session key (*K*_*i*_) for each group *S*_*i*_ and a ciphertext *𝒞* for all groups. The broadcaster entity makes ciphertext *𝒞* available publicly and session keys (*K*_*i*_)_*i*=1_^*m*^ are kept secret in the system. To recover a ciphertext *C*_*i*_ for plaintext message *M*, one must have session key *K*_*i*_. This scheme is based on symmetric-key encryption algorithm. If *S*_*i*_=*ϕ* (null set) then the broadcaster entity sets *K*_*i*_=⊥.
4. *K*_*i*_ ← Dec(Param, *d*_*u*_, *𝒞*, {*S*_*i*_}_*i*=1_^*m*^): a subscribed user *u* ∈ *S*_*i*_ retrieves his/her session key *K*_*i*_ corresponding to group *S*_*i*_ using *d*_*u*_, Param, *𝒞* and (*S*_1_, *S*_2_,…, *S*_*m*_).

Correctness-The MCBE scheme holds correctly if, for a legitimate user *u*⊆*S*_*i*_, the session key *K*_*i*_ can be fetched from ciphertext *𝒞* correctly.

## 3. Conversion from Type-I Pairing to Type-III Pairing

Many novel applications have been constructed using pairing-based cryptographic protocols that are based on bilinear pairing map $\hat{e}:{𝔾}_{1}\times {𝔾}_{2}⟶{𝔾}_{t}$, where *𝔾*_1_ and *𝔾*_2_ are candidate prime order groups of a meticulously chosen elliptic curve *ℰ* over a finite field *𝔽*_*q*_, and *𝔾*_*t*_ is a subgroup of finite field *𝔽*_*q*_. Bilinear pairing is realizable from Weil, Tate, and other optimal pairings of elliptic curves [31].

Bilinear maps are studied extensively and have been efficiently implemented in past decades [35]. Bilinear pairings are broadly categorized into the following:

1. Asymmetric pairing
2. Symmetric pairing
3. Composite order pairing

An *asymmetric pairing* is a general bilinear map that efficiently computes $\hat{e}:{𝔾}_{1}\times {𝔾}_{2}⟶{𝔾}_{t}$, where *𝔾*_1_ is a *q*-prime order group of points of an elliptic curve over a finite field *𝔽*_*q*_ and *𝔾*_2_ is also of the same prime order group of that elliptic curve over an extension field of *𝔽*_*q*_. When the domains of bilinear map $\hat{e}$ are identical, such a pairing function $\hat{e}$ is referred to as a *symmetric pairing*. The third type of pairing is *composite order pairing* [36], where *𝔾*_1_ is of composite order. The provision of additional flexibility makes computation of composite pairing slower. Waters' dual encryption system [37] was first constructed using composite order groups and in his later work composite order identity-based encryption is transformed and constructed using prime order bilinear pairing in asymmetric setting. The conversion from composite order bilinear pairing to prime order bilinear pairing was due to efficiency consideration. Studies have recommended that asymmetric pairings are faster and compact from the implementation viewpoint. Asymmetric bilinear pairings have the possibility to reduce the size of group in ciphertext and keys (public key and private key). There have been enormous cryptographic constructions realized on bilinear maps $\hat{e}:𝔾\times 𝔾⟶{𝔾}_{T}$. Here, multichannel broadcast encryption (MCBE) cryptographic primitive is built from bilinear pairings. Asymmetric bilinear pairings are further categorized into Type-II and Type-III bilinear pairings. In case of Type-II setting, there exists an efficiently computable isomorphism from group *𝔾*_1_ to group *𝔾*_2_ or vice versa, whereas in the Type-III pairing no such kind of isomorphism is known. Previous work has shown that the Type-III setting is the most efficient (among all pairing types) form in terms of operations required and security.

The following steps show the conversion from Type-I MCBE to Type-III MCBE for all the four algorithms (Setup, KeyGen, Enc, Dec).

(1) **Setup**(1^*λ*^, *n*):
  (a) Randomly select *α* ∈ *ℤ*_*q*_.
  (b) Given $\hat{e}:{𝔾}_{1}\times {𝔾}_{2}⟶{𝔾}_{T}$, *𝔾*_1_=〈*P*〉, *𝔾*_2_=〈*Q*〉.
  (c) Select *n* random scalars (*x*_1_, *x*_2_,…, *x*_*n*_).
  (d) Evaluate *X*_1_=*Q*^*x*_1_^, *X*_2_=*Q*^*x*_2_^,…, *X*_*n*_=*Q*^*x*_*n*_^.
  (e) Set $\text{Param}=\left(P,Q,{\overset{⟶}{Y}}_{P,\alpha ,n},{\overset{⟶}{Y}}_{Q,\alpha ,n},\left(X_{1},X_{2},\ldots ,X_{n}\right)\right)$.
(2) **Keygen**(MSK, PK)**:**
  (a) Randomly select *γ* ∈ *ℤ*_*q*_ and set MSK=(*γ*, *α*, (*x*_1_, *x*_2_,…, *x*_*n*_)).
  (b) Set *ν*=*P*^*γ*^ ∈ *𝔾*_1_.
  (c) Set public key PK=(Param, *γP*, *γQ*).
  (d) Secret key SK for users *i*=1,2,…, *n* is computed as *d*_*i*_=*ν*^*α*^*i*^^.
(3) **Encrypt**(*S*_1_, *S*_2_,…, *S*_*m*_, Param)⟶(*𝒞*, *K*_1_, *K*_2_,…, *K*_*m*_):
  (a) Select a random scalar $r\overset{\left[R\right]}{\leftarrow }{\mathbb{Z}}_{q}$.
  (b) Set $K_{k}=\hat{e}{\left(P_{n},Q_{1}\right)}^{\left(r+\sum_{j\in S_{k}}x_{j}\right)}$.
  (c) Evaluate
    (1) $$\begin{array}{c} C_{1}=Q^{r},\\ C_{2}=\prod_{k=1}^{m}{\left(\nu \cdot \underset{j\in S_{k}}{\prod }P_{n+1-j}\right)}^{\left(r+\sum_{j\in S_{k}}x_{j}\right)}. \end{array}$$
  (d) Set *𝒞*=(*𝒞*_1_, *𝒞*_2_).
  (e) Communicate *𝒞*.
(4) **Decrypt**(*S*_1_, *S*_2_,…, *S*_*m*_, *𝒞*, *d*_*i*_, *i*)⟶*K*_*k*_: if *i* ∈ *S*_*k*_ then compute

(2) $$\begin{array}{c} K_{k}=\frac{\hat{e}\left(Q_{i},C_{2}\right)}{\hat{e}\left(d_{i}\cdot \prod_{j\neq i,j\in S_{k}}P_{n+1-j+i},C_{1}\cdot \prod_{j\in S_{k}}X_{j}\right)}\cdot \frac{1}{\hat{e}\left(d_{i}\cdot \prod_{j\in S_{l}}P_{n+1-j+i},C_{1}\cdot \prod_{j\in S_{l}}X_{j}\right)}. \end{array}$$

Substituting the value of *𝒞*_1_

(3) $$\begin{array}{c} =\frac{\hat{e}\left(Q_{i},C_{2}\right)}{\hat{e}\left(d_{i}\cdot \prod_{j\neq i,j\in S_{k}}P_{n+1-j+i}\right),Q^{r}\cdot \prod_{j\in S_{k}}X_{j}}\cdot \frac{1}{\prod_{l=1l\neq k}\hat{e}\left(d_{i}\cdot \prod_{j\neq i,j\in S_{k}}P_{n+1-j+i},Q^{r}\cdot \prod_{j\in S_{k}}X_{j}\right)}\\ =\frac{\hat{e}\left(Q^{\alpha^{i}},\prod_{l=1}^{m}{\left(\nu \cdot \prod_{j\in S_{l}}P_{n+1-j}\right)}^{\left(r+\sum_{j\in S_{l}}x_{j}\right)}\right)}{\hat{e}\left(\nu^{\alpha^{i}}\cdot {\left(\prod_{j\neq i,j\in S_{k}}P_{n+1-j+i}\right)}^{\alpha^{i}},Q^{\left(r+\sum_{j\in S_{l}}x_{j}\right)}\right)}\cdot \frac{1}{\prod_{l=1,l\neq k}^{l=m}\hat{e}\left(\nu^{\alpha^{i}}\cdot \prod_{j\in S_{l}}P_{n+1-j+i},Q^{\left(r+\sum_{j\in S_{l}}x_{j}\right)}\right)}\\ =\frac{\hat{e}{\left(Q^{\alpha^{i}},\nu \cdot \prod_{j\in S_{l}}P_{n+1-j+i}\right)}^{\left(r+\sum_{j\in S_{k}}x_{j}\right)}}{\hat{e}\left({\left(\nu \cdot \prod_{j\neq i,j\in S_{k}}P_{n+1-j+i}\right)}^{\alpha^{i}},Q^{\left(r+\sum_{j\in S_{k}}x_{j}\right)}\right)}\cdot \prod_{l=1,l=k}^{m}\frac{\hat{e}{\left(Q^{\alpha^{i}},\nu \cdot \prod_{j\in S_{l}}P_{n+1-j+i}\right)}^{\left(r+\sum_{j\in S_{l}}x_{j}\right)}}{\hat{e}\left({\left(\nu \cdot \prod_{j\neq i,j\in S_{l}}P_{n+1-j+i}\right)}^{\alpha^{i}},Q^{\left(r+\sum_{j\in S_{l}}x_{j}\right)}\right)}\\ =\frac{\hat{e}\left({\left(\nu \cdot \prod_{j\in S_{k}}P_{n+1-j+i}\right)}^{\alpha^{i}},Q^{\left(r+\sum_{j\in S_{k}}x_{j}\right)}\right)}{\hat{e}\left({\left(\nu \cdot \prod_{j\neq i,j\in S_{k}}P_{n+1-j+i}\right)}^{\alpha^{i}},Q^{\left(r+\sum_{j\in S_{k}}x_{j}\right)}\right)}\cdot \prod_{l=1,l=k}^{m}\frac{\hat{e}{\left(\alpha^{i}Q,\nu \cdot \prod_{j\in S_{l}}P_{n+1-j+i}\right)}^{\left(r+\sum_{j\in S_{l}}x_{j}\right)}}{\hat{e}\left({\left(\nu \cdot \prod_{j\neq i,j\in S_{k}}P_{n+1-j+i}\right)}^{\alpha^{i}},Q^{\left(r+\sum_{j\in S_{l}}x_{j}\right)}\right)}\\ =\hat{e}\left(P_{n+1-i}^{\alpha^{i}},Q^{\left(r+\sum_{j\in S_{k}}x_{j}\right)}\right)\\ =\hat{e}\left(P_{n+1},Q^{\left(r+\sum_{j\in S_{k}}x_{j}\right)}\right)\\ K_{k}=\hat{e}{\left(P_{n},Q_{1}\right)}^{\left(r+\sum_{j\in S_{k}}x_{j}\right)}. \end{array}$$

## 4. Security Model

**Figure 4:**
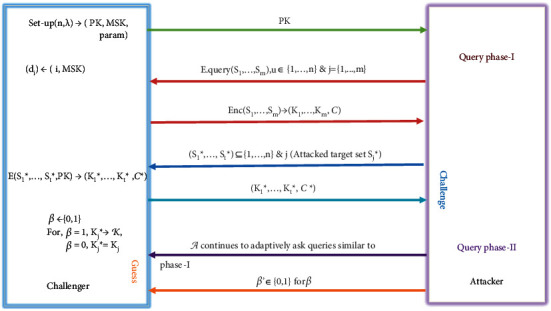
Security model of MCBE.

We also define the formal framework of security of MCBE scheme in asymmetric-pairing setting by the following game between the *adversarial algorithm𝒜* and a *simulator algorithm𝒞* in a real or random setting [38], as shown in Figure 4.

(1) **Setup**: the simulator algorithm *𝒞* runs the *Setup* algorithm and outputs Param, MSK, and encryption key (PK).
(2) **Query Phase-I**: *𝒜* adaptively asks queries to *𝒞* which is also known as a Challenger. For some *i*-th user *u*_*i*_, where *i*=(1,2,…, *n*), *𝒞* sends the decryption keys to *𝒜*. In response to the encryption query, *𝒞* evaluates Enc(*S*_1_, *S*_2_,…, *S*_*m*_, Param) to produce (*K*_1_, *K*_2_,…, *K*_*m*_, *𝒞*) as output.
(3) **Challenge**: at this stage, *𝒜* forwards the challenge set (*S*_1_^*∗*^, *S*_2_^*∗*^,…, *S*_*t*_^*∗*^), where each *S*_*i*_^*∗*^⊆{1,2,…, *n*} for *i*=1,2,…, *t*, as well as a target set *S*_*j*_^*∗*^, where *j* ∈ {1,2,…, *t*}, to *𝒞*. In response to this, *𝒞* forwards (*K*_1_^*∗*^, *K*_2_^*∗*^,…, *K*_*t*_^*∗*^, *𝒞*^*∗*^). Then, *𝒞* selects random $\beta \overset{\left[R\right]}{\leftarrow }\left\{0,1\right\}$. Depending on the value of *β*, *𝒞* replies with the following response ℛ:
  (4) $$\begin{array}{c} ℛ=\left\{\begin{array}{c} K_{j}^{*}\text{ is real key},\beta =0\\ K_{j}^{*}\text{ is random},\beta =1 \end{array}\right.. \end{array}$$
(4) **Query Phase-II**: *𝒜* continuously asks queries similar to *Query Phase-I.*
(5) **Guess**: now, *𝒜* eventually returns decision bit *β*′ ∈ {0,1} for *β*.

(5) $$\begin{array}{c} \mathrm{Ad}v_{\text{MCBE}}^{A}=\mathrm{Pr}\left[1\leftarrow A|\beta =1\right]-\text{Pr}\left[1\leftarrow A|\beta =0\right]. \end{array}$$

**Theorem 1 .**

*The*MCBE*scheme in asymmetric setting is selectively secure under DBDHE assumption if it holds in*(*𝔾*_1_, *𝔾*_2_)*. For maximumnnumber of legitimate users, ***Adv**(*τ*, *q*) ≤ 2 × **Adv**^*b*  *dh*  *e*^(*τ*′, *n*)*, forτ*′ ≤ *τ*+(*xn*+*nq*)*T*_*e*_*, whereT*_*e*_*denotes time complexity for exponentiation computation andmrepresents maximum number of available channels in the system.*

**Proof**

Let us consider that there exists Probabilistic Polynomial Time (PPT) algorithm, *𝒜*, such that **Adv**_MCBE_^*𝒜*,*n*^ > 1/2+*ϵ* for an MCBE system. We build a simulator algorithm *𝒞* that has advantage in solving the DBDHE problem in (*𝔾*_1_, *𝔾*_2_). Algorithm *𝒞* takes as input a challenge $\left(P,Q,\;ℋ,{\overset{⟶}{Y}}_{P,\alpha ,n},{\overset{⟶}{Y}}_{Q,\alpha ,n},D\right)$, where *D* is either $\hat{e}\left(P_{n+1},\;ℋ\right)\in {𝔾}_{T}$ or a random element ⊥∈*𝔾*_*T*_.

1. Setup

1. *𝒞* generates global public parameters and secret keys *d*_*i*_ for *i* ∈ *S*_*k*_. It selects a random scalar *r* ∈ *ℤ*_*q*_.
2. Set *h*=*Q*^*r*^⇒*h*_*i*_=*Q*_*i*_^*r*^ for all *i*={1,2 …, *n*}.
3. Select random scalars *x*_*i*_ ∈ *ℤ*_*q*_ for *i*={1,2,…, *η*,…, *n*} and compute *X*_*i*_=*Q*^*x*_*i*_^.
4. Choose a random index *η* ∈ *S*_*k*_.
5. *X*_*η*_=ℋ/∏_*i*∈*S*_*k*_*i*∉*η*_*X*_*i*_=*Q*^*x*_*η*_^. All scalars are known except *x*_*η*_.
6. *𝒞* provides adversary the global public parameters: $\text{Param}=\left(P,Q,{\overset{⟶}{Y}}_{P,\alpha ,n},{\overset{⟶}{Y}}_{Q,\alpha ,n},{\left\{X_{i}\right\}}_{i=1}^{n}\right)$.
7. *𝒞* performs computation of secret decryption keys *d*_*i*_ except for *i* ∈ *S*_*k*_.
8. Select a random legitimate user *u* ∈ *ℤ*_*q*_ and define

(6) $$\begin{array}{c} \nu^{de\;f=}\frac{Q^{u}}{\left(\prod_{j\in S_{k}}P_{n+1-j}\right)}\\ d_{i}^{de\;f=}\frac{Q_{i}^{u}}{\left(\prod_{j\in S_{k}}P_{n+1-j+1}\right)}\\ ={\left(\frac{Q^{u}}{\prod_{j\in S_{k}}n+1-j}\right)}^{\alpha^{i}}. \end{array}$$

Substituting the value of *ν*, we get

(7) $$\begin{array}{c} d_{i}=\nu^{\alpha^{i}}=\nu_{i}. \end{array}$$

Moreover, since *d*_*i*_=*ν*^*α*^*i*^^, it satisfies the specification parameters of the construction.

(2) Challenge

(1) Challenge C is simulator algorithm. while *𝒞*=(*𝒞*_1_, *𝒞*_2_) is part of header, denotes cipher text both C and *𝒞*.
(2) *P*_*j*∈*S*_*l*__=∏_*j*∈*S*_*l*__*P*_*n*+1−*j*_ and *P*_*j*∈*S*_*k*__=∏_*j*∈*S*_*k*__*P*_*n*+1−*j*_.
Evaluate *𝒞*_2_ as
  (8) $$\begin{array}{c} C_{2}=\left(h^{u}\cdot {ℋ}^{u}\right)+\prod_{l=1,l\neq k}^{m}\left(h^{u}\cdot \left(\frac{P_{j\in S_{l}}}{P_{j\in S_{k}}}\right)\cdot {\left(\nu \cdot P_{j\in S_{l}}\right)}^{\sum_{j\in S_{l}}x_{j}}\right)\\ {\left(Q^{u}\right)}^{r+\sum_{j\in S_{k}}x_{j}}+\prod_{l=1,l\neq k}^{m}\left(Q^{ru}\left(\frac{P_{j\in S_{l}}}{P_{j\in S_{k}}}\right)\cdot {\left(\nu \cdot P_{j\in S_{l}}\right)}^{\sum_{j\in S_{l}}x_{j}}\right)\\ ={\left(Q^{u}\right)}^{r+\sum_{j\in S_{k}}x_{j}}+\prod_{l=1,l\neq k}^{m}\left({\left(\frac{Q^{u}}{P_{j\in S_{k}}}\right)}^{r}\cdot {\left(P_{j\in S_{l}}\right)}^{r}\cdot {\left(\nu \cdot P_{j\in S_{l}}\right)}^{\sum_{j\in S_{l}}x_{j}}\right). \end{array}$$
Substituting the value of *Q*^*u*^, we get
  (9) $$\begin{array}{c} ={\left(\nu P_{j\in S_{k}}\right)}^{r+\sum_{j\in S_{k}}x_{j}}+\overset{m}{\underset{l=1,l\neq k}{\prod }}\left({\left(\frac{\nu P_{j\in S_{k}}}{P_{j\in S_{k}}}\right)}^{r}{\left(P_{j\in S_{l}}\right)}^{r}{\left(\nu P_{j\in S_{l}}\right)}^{\sum_{j\in S_{l}}x_{j}}\right)\\ ={\left(\nu \underset{j\in S_{k}}{\prod }P_{n+1-j}\right)}^{r+\sum_{j\in S_{k}}x_{j}}+\overset{m}{\underset{l=1,l\neq k}{\prod }}{\left(\nu \underset{j\in S_{l}}{\prod }P_{n+1-j}\right)}^{r}{\left(\nu \underset{j\in S_{l}}{\prod }P_{n+1-j}\right)}^{\sum_{j\in S_{l}}x_{j}}\\ \overset{m}{\underset{l=1}{\prod }}{\left(\nu \cdot \underset{j\in S_{l}}{\prod }P_{n+1-j}\right)}^{r+\sum_{j\in S_{l}}x_{j}}. \end{array}$$
The following notations were used: *P*_*n*+1−*j*_^*r*^=*h*_*n*+1−*j*_ and ℋ=*Q*^∑_*j*∈*S*_*i*__*x*_*j*_^.
(3) Set *𝒞*=(*𝒞*_1_, *𝒞*_2_).
(4)
$D=\hat{e}\left(P_{n+1},ℋ\right)$ and $K_{k}=\hat{e}{\left(Q_{1},P_{n}\right)}^{r+\sum_{j\in S_{k}}x_{j}}$.
(5) Thus, to generate session keys, *𝒞* computes, for all *i* ≠ *k*,
  (10) $$\begin{array}{c} K_{i}=\hat{e}{\left(P_{n+1},Q\right)}^{\sum_{j\in S_{i}}x_{j}}\cdot \hat{e}\left(P_{n+1},Q^{r}\right)\\ =\hat{e}{\left(P_{n+1},Q\right)}^{r+\sum_{j\in S_{i}}x_{j}}, \end{array}$$
and sets
  (11) $$\begin{array}{c} K_{k}=D\cdot \hat{e}\left(P_{n+1},Q^{r}\right). \end{array}$$
(6) *𝒞* produces the output (*𝒞*, {*K*_*i*_}_*i*=1_^*m*^) as a challenge to adversarial algorithm *𝒜*.
(7) If *D* is the correct value, then

(12) $$\begin{array}{c} K_{k}=\hat{e}\left(P_{n+1},ℋ\right)\cdot \hat{e}\left(P_{n+1},Q^{r}\right). \end{array}$$

Substituting the value of ℋ, we get

(13) $$\begin{array}{c} K_{k}=\hat{e}\left(P_{n+1},Q^{\sum_{j\in S_{i}}x_{j}}\right)\cdot \hat{e}\left(P_{n+1},Q^{r}\right)\\ \hat{e}{\left(P_{n+1},Q\right)}^{r+\sum_{j\in S_{i}}x_{j}}. \end{array}$$

If the value of element *D* is random, then *K*_*k*_ produces ⊥ as output.

(3) Guess

*𝒜* outputs a guess bit *β*′ for *β*. *𝒜* wins the game if *β*′=*β*. *𝒞* produces the output ℛ:

(14) $$\begin{array}{c} ℛ=\left\{\begin{array}{c} 0,\beta^{\prime }=\beta \\ 1,\beta^{\prime }\neq \beta \end{array}\right.. \end{array}$$

ℛ=0 represents $D=\hat{e}\left(P_{n+1},\;ℋ\right)$; otherwise, when ℛ=1, *D*=⊥.

*𝒜*'s advantage in breaking the security of the MCBE in asymmetric setting is defined in terms of the fact that the probability of occurrence of the event that *β*′=*β* in the mentioned game is evaluated as **Adv**_*MCBE*_^*𝒜*^=|Pr[*β*′=*β*] − 1/2|

## 5. Result and Analysis

We have presented multichannel broadcast encryption (MCBE) scheme in asymmetric-pairing setting. The number of scalars, which have been used in the construction of MCBE (in symmetric-pairing setting), is analyzed. The following observations were made:

1. Param uses scalars (*x*_1_, *x*_2_,…, *x*_*n*_) and *α*. MSK uses scalars (*x*_1_, *x*_2_,…, *x*_*n*_), *α*, and *γ*.
2. Encrypt algorithm uses scalars *r*, *γ*, *m* (no. of groups).
3. Ciphertext uses *r*, *γ* scalars.
4. Param, MSK, EK, and ciphertext consist of elements of group *𝔾*_1_.
5. $T=\hat{e}\left(P_{n+1},G\right)\in {𝔾}_{T}$ [4].

Based on the above points, we have attempted a transformation of the scheme into Type-III pairing setting. The MCBE in asymmetric setting has the following points:

1. Param uses (2*n* − 1) elements of *𝔾*_1_ and (3*n* − 1) elements of *𝔾*_2_. MSK uses scalars (*x*_1_, *x*_2_,…, *x*_*n*_), *α*, and *γ*.
2. Enc algorithm uses scalar *r* only.
3. Ciphertext uses *r* and *γ*.
4. Param and ciphertext consist of elements of groups *𝔾*_1_ and *𝔾*_2_.
5. MSK and SK consist of elements of group *𝔾*_2_.
6. PK consists of elements of (*𝔾*_1_, *𝔾*_2_).
7. $D=\hat{e}\left(P_{n+1},ℋ\right)\in {𝔾}_{T}$.

A comparison of features of MCBE scheme based on various complexity assumptions is shown in Table 2. The rows # Param, # PK, # SK, # CT, # EK, and # MSK represent the numbers of group elements in public parameter, public key, secret key, ciphertext, encryption key, and master secret key, respectively.

Group *𝔾*_1_ consists of two parameters, Param and ciphertext. On the other hand, MSK and decryption keys (SK) are elements of *𝔾*_2_. All MCBE schemes appearing in literature are included in Table 3.

Based on Tables 2 and 3, the following has been observed:

- The proposed scheme uses asymmetric pairings, whereas the rest of the schemes use asymmetric pairings. So, one could have |*𝔾*_2_| > |*𝔾*_1_|, which in turn leads to the smaller size ciphertext, reduced storage space, and enhanced performance [31].
- As we have taken two group elements *𝔾*_1_ and *𝔾*_2_ and achieved compact size ciphertext which is the most important design consideration of broadcast encryption schemes, the public parameter size has increased in the proposed method. It is the limitation of this work. The public parameter is independent of the number of channels and users need to download it once. It does not increase any communication overhead.

## 6. Conclusion and Future Work

We have proposed an efficient and secure method for data sharing using asymmetric pairing (Type-III) with compact size ciphertext in public-key setting to enable rapid learning in healthcare environment. Our construction serves as an efficient solution for various practical data sharing applications such as healthcare environment, distribution of consumer product licence, and collaborative sharing to enable learning. Our construction is collusion-resistant and the security of the scheme is based on standard hardness assumption. We have demonstrated how this construction is modified to symmetric pairing to achieve compact size ciphertext. The analysis and result establish that the proposed scheme outperforms other existing schemes in terms of performance, storage, and transmission cost. The proposed method offers the same level of security with reduced memory requirement. Reducing the size of public parameter as well as constructing the traitor tracing system for this scheme is left as open problem.

## Figures and Tables

**Table 1:** Notations.

Notation	Description
*λ*	Security parameter
Param	Public parameter
*𝔾*_1_, *𝔾*_2_, *𝔾*_*T*_	Cyclic groups
*P*, *Q*	Generator element
${\overset{⟶}{Y}}_{N,\alpha ,l}$	Vector set
MSK	Master secret key
$\hat{e}$	Pairing function
PK	Public key
SK	Decryption key
*𝒞*, *𝒜*	Algorithms

**Table 2:** A comparative summary of various variants of MCBE schemes.

Parameters	[4]	[22]	[16]	[24]	[23]	Proposed
|Param|	(3*n* − 1)|*𝔾*|	(2*n*+*m*)|*𝔾*|	(2*n*+*m*+3)|*𝔾*|	(*n*+mn+1)|*𝔾*_1_|	(3*n*+*m*+3)|*𝔾*|	(2*n*+*m* − 1)|*𝔾*_1_|
				*m*|*𝔾*_*T*_|		(2*n* − 1)|*𝔾*_*𝕋*_|

|SK|	1|*𝔾*|	1|*𝔾*|	1|*𝔾*|	1|*𝔾*_1_|	2|*𝔾*|	1|*𝔾*_1_|
|CT|	2|*𝔾*|	2|*𝔾*|	2|*𝔾*|	1|*𝔾*_1_|	2|*𝔾*|	1|*𝔾*_1_|
Public key	No	No	Yes	Yes	Yes	Yes
Security	CPA	CPA	CPA	CPA	CPA	CPA
Hardness assumption	*n*-BDHE	*n*-BDHE	DBDHE-sum	GDDHE	*n*-BDHE	*n*-BDHE
Algebraic construction	Pairing based	Pairing based	Pairing based	Pairing based	Pairing based	Pairing based
Pairing type	Type-I	Type-I	Type-I	Type-III	Type-I	Type-III
Random oracle	No	No	No	Yes	No	No

**Table 3:** A comparison of various symmetric- and asymmetric-pairing-based MCBE schemes.

Schemes	# Param		# CT		# MSK		# SK		Pairing
	*𝔾*_1_	*𝔾*_*T*_	*𝔾*_1_	*𝔾*_2_	*𝔾*_1_	*ℤ*_*q*_	*𝔾*_1_	*ℤ*_*q*_	
[4]	3*n* − 1	−	2	−	2	*n*+2	2	*n*+1	SP
[22]	2*n*+*m*	−	2	−	2	−	1	2	SP
[16]	2*n*+*m*+3	−	2	−	2	*n*	1	2	SP
[24]	*n*+*mn*+1	m	1	1	1	*m*+1	1	*m*	AP
[23]	3*n*+*m*+3	−	2	−	2	−	2	−	SP
Proposed	2*n*+*m* − 1	2*n* − 1	1	1	−	*n*+2	1	2	AP

SP: symmetric pairing; AP: asymmetric pairing.

## Data Availability

The data that support the findings of this study are available from the corresponding author upon request. The dataset is not required for this study.
